# Synergistic Deformation of Ferrite/Martensite Laminates Brings High Strength and Good Ductility in Dual-Phase Steel

**DOI:** 10.3390/ma18174198

**Published:** 2025-09-07

**Authors:** Lijuan Zhang, Pengzhan Cai, Ling Zhang, Ziyong Hou, Guilin Wu

**Affiliations:** 1 Chongqing Aerospace Polytechnic, Chongqing 400021, China; zhanglijuan@cqht.edu.cn; 2 International Joint Laboratory for Light Alloys (MOE), College of Materials Science and Engineering, Chongqing University, Chongqing 400044, China; pengzhancai@cqu.edu.cn (P.C.);; 3 Sichuan Academy of Aerospace Technology, Chengdu 610100, China; 4 State Key Laboratory of Mechanical Transmission for Advanced Equipment, Chongqing University, Chongqing 400044, China; 5 Institute of Advanced Interdisciplinary Studies, Chongqing University, Chongqing 400044, China

**Keywords:** dual-phase steel, mechanical properties, nano-indentation, micropillar compression, deformation mechanism

## Abstract

A low-carbon ferrite/martensite-laminated 0.1C5Mn3Al dual-phase steel was hot-rolled to an engineering strain of 98%, and a tensile strength of 1277 ± 44 MPa and a total elongation of 11.8 ± 0.4% was obtained in the steel. Hot-rolling induces a laminated/layered structure characterized by alternating ferrite phases and martensite phases distributed perpendicular to the rolling direction. A deformation mechanism was evaluated using nano-indentation and in situ compression of micropillars in a scanning electron microscope. The excellent mechanical properties of the steel are attributed to the refinement of ferrite/martensite layers and ultra-fine martensite laths. The synergistic deformation of the ferrite and martensite laminates provides the steel with a good combination of high strength and tensile elongation.

## 1. Introduction

Metals and alloys with high strength and good ductility are critical for structural materials employed in many engineering applications. Laminated materials have both high strength and ductility, and therefore their microstructure manipulation and mechanical properties have been studied recently [1,2,3,4,5,6]. Layered and laminated materials exhibit a synergistic enhancement of strength and ductility through the combined action of hetero-deformation-induced (HDI) strengthening and the formation of dispersive microscopic strain bands [1]. N. Koga discussed the influence of layer thickness on deformation and fracture behavior [2]. The influence of microstructural factors on mechanical properties of layered and laminated materials is a focal point of extensive investigation [3,4,5,6]. Typically, a low-carbon laminated dual-phase steel is designed by using Mn and Al elements to stabilize austenite at high temperatures and suppress the growth of lamellae [7,8,9]. In our previous studies, after a medium strain (63%) hot-rolling, a yield stress of 789 MPa and a total elongation of about 25% were obtained. Cold deformation of the hot-rolled material could increase the strength to 1290 MPa, but the elongation was dramatically decreased to only 3% [10,11]. Unfortunately, further heat treatment did not produce better properties than the hot-rolled one. The deteriorated elongation was attributed to microstructural change during annealing, indicating that cold deformation followed by heat treatment is unfavorable to further improve the mechanical properties of this dual-phase steel. Based on this information, we designed a hot-rolling method with a larger strain of 98% and found that the strength of the low-carbon 0.1C5Mn3Al dual-phase steel can be improved to 1277 ± 44 MPa with an elongation of 11.8 ± 0.4%, resulting in a superior approach to tailor the mechanical properties of dual-phase steel, which is similar to that reported by Wang et al. [12]. To understand the deformation mechanism, nano-indentation and in situ micropillar compression tests in scanning electron microscopy were performed in the ferrite phase, martensite phase, and dual-phase interfaces to reveal the unique deformation mechanism of the experimental steel.

## 2. Materials and Methods

The 0.1C5Mn3Al dual-phase steel with a measured composition of 0.11 wt% C, 4.91 wt% Mn, 3.11 wt% Al, and 0.21 wt% Si was used in this study. The steel was fabricated in a vacuum induction furnace and cast into a 50 kg ingot. The ingot was hot-forged into a billet with a length of 50 mm. After homogenizing at 1200 °C for 2 h, the billet was hot-rolled to a rod with a final diameter of 8 mm at a finishing temperature of approx. 950 °C, and followed by air cooling to room temperature. The total engineering strain of hot-rolling is 98%, and thus the sample is shorted as sample HR-98%. For comparison, the former-mentioned 63% hot-rolled sample was further cold-forged to 60%, corresponding to a total deformation strain of 98%, with the compression direction of forge parallel to the normal direction of rolling, and the forged sample was employed in this study (shorted as HR + CF). Detailed information can be found in Ref. [10]. Dog-bone-shaped tensile samples with a gauge length of 8 mm, a width of 2 mm, and a thickness of 1 mm were used. Tensile tests were conducted at ambient temperature with a strain rate of 10^−3^ s^−1^. Nano-indentation measurements were performed using a Hysitron Triboindenter (Minneapolis, MN, USA) with a Berkovich indenter under a load-controlled model. A 5 mN peak load was used with loading and unloading rates of 500 μN/s. Samples for microstructural analysis were polished and etched in 2% natal for 5 s. Micropillars were fabricated by using focused ion beam (FIB) in a Zeiss Auriga dual-beam station. In situ compression tests were performed by Hysitron PI88 PicoIndenter with a 20 μm diameter flat punch in the Zeiss Auriga dual-beam station. All micropillars were compressed to a nominal strain of about 20% under displacement control mode with a speed of 4 nm/s. The diameter of the micropillar’s top surface and the initial height were used to calculate the engineering stress and strain. Small pillars (2 μm) containing single phases or one interface and large (7 μm) pillars containing two interfaces were used. Thin foils of the deformed micropillars for transmission electron microscope (TEM) observation were prepared by the lift-out method in FIB.

## 3. Results and Discussion

**Figure 1:**
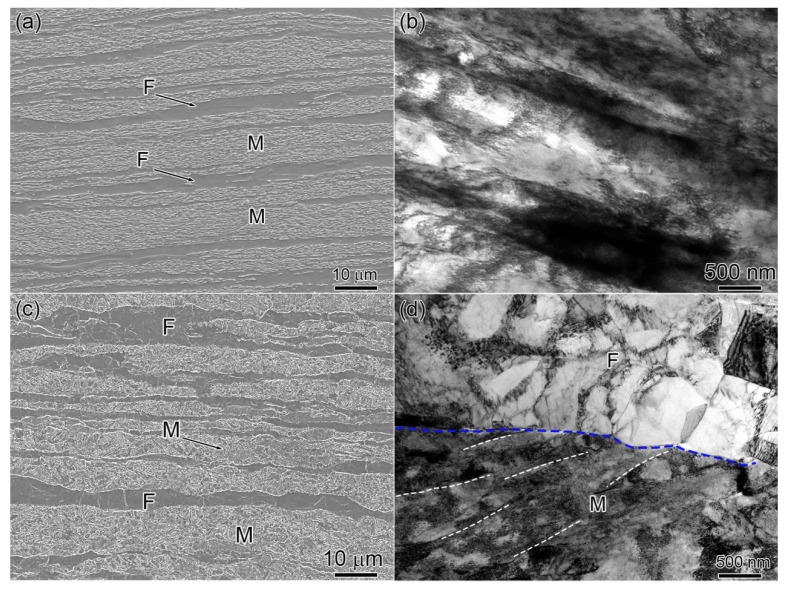
SEM secondary electron image and TEM bright field image of (**a**), (**b**) HR + CF, (**c**), and (**d**) HR-98%.

Typical microstructures of HR-98% and HR + CF are shown in Figure 1. Both samples show a laminated/layered structure characterized by a ferrite phase and martensite phase alternatively distributed perpendicular to the rolling direction (RD). (Note: the RD of the sample HR + CF represents the rolling direction of the hot-rolling step.) The volume fraction of martensite (VM) of HR-98% and HR + CF are both close to 60%. As shown in Figure 1a, the average layer thicknesses of the martensite and ferrite phases of the HR + CF sample are 2.6 ± 2.4 μm and 2.2 ± 8.1 μm, respectively. From Figure 1b, it can be seen that the HR + CF sample shows a typical, severely deformed laminated structure and the average thickness is approx. 770 nm. On the other hand, as shown in Figure 1c, the layer thickness of the HR-98% sample are 3.6 ± 2.7 μm for martensite and 2.3 ± 1.9 μm for ferrite, respectively. Figure 1d shows the ferrite layers consist of equiaxed dislocation cells and sub-grains with an average size of approximately 540 nm, while the martensite lamellae are characterized by thin martensite laths with an average thickness of approximately 200 nm. For dual-phase steels with retained austenite to produce a transformation-induced plasticity (TRIP) effect during deformation, Ma et al. developed a 1200 MPa grade dual-phase steel through the addition of V and Nb. 1200 Mpa [13]. A new 2.4 GPa extra-high strength steel with good ductility and high toughness was also designed using the transformation-induced plasticity TRIP effect [14]. However, no retained austenite was detected in the 0.1C5Mn3Al dual-phase steel, and the deformation mechanism is significantly different. To understand the deformation mechanism, nano-indentation and in situ micropillar compression tests in scanning electron microscopy were performed.

**Figure 2:**
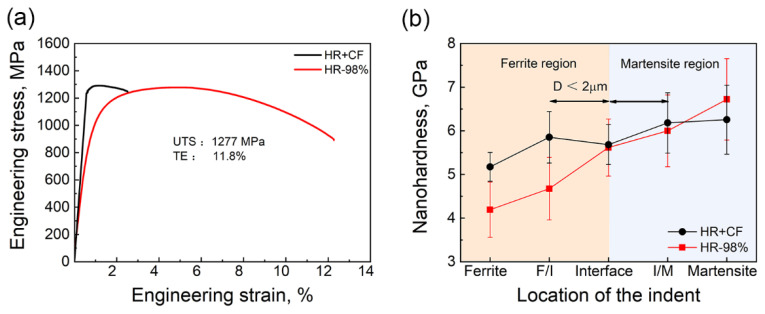
(**a**) Engineering stress–strain curves and (**b**) average nanohardness at various locations.

Figure 2a shows the engineering stress–strain curves of the two samples. It is seen that the ultimate tensile strength (UTS) of the HR + CF sample is 1290 ± 40 MPa and the total elongation (TE) is 2.5 ± 0.5%. However, the HR-98% sample has a UTS of 1277 ± 44 MPa, a yield strength (YS) of 965 ± 36 MPa, and a total elongation (TE) of 11.8 ± 0.4%, much superior to the HR + CF sample, indicating that much better mechanical properties can be obtained by large-strain hot deformation than cold deformation. The nanohardness of the two samples are compared in Figure 2b. It is found that the nanohardness for the ferrite phase (both in the interiors and close to the interface) of the HR + CF sample is larger than those of the HR-98% sample. This is consistent with the microstructures where the ferrite phases in the HR + CF sample are full of deformation-induced dislocations while those in the HR-98% sample are equiaxed sub-grains. The nanohardness of the interface (can be treated as average nanohardness of the two phases) and the martensite phase close to the interface of HR-98% is close to that of the HR + CF sample. On the other hand, the nanohardness of the martensite in HR-98% is slightly higher than that in HR + CF, which indicates that increasing the hot deformation strain is useful to improve the hardness of the transformed martensite [15,16,17]. According to the nano-indentation tests, the high strength of the HR sample can be attributed to the hard martensite phase though the reason for the good ductility is not clear. To understand the reason, in situ micropillar compression tests in scanning electron microscopy were performed not only in single ferritic or martensitic phases, but also including dual-phase interfaces.

**Figure 3:**
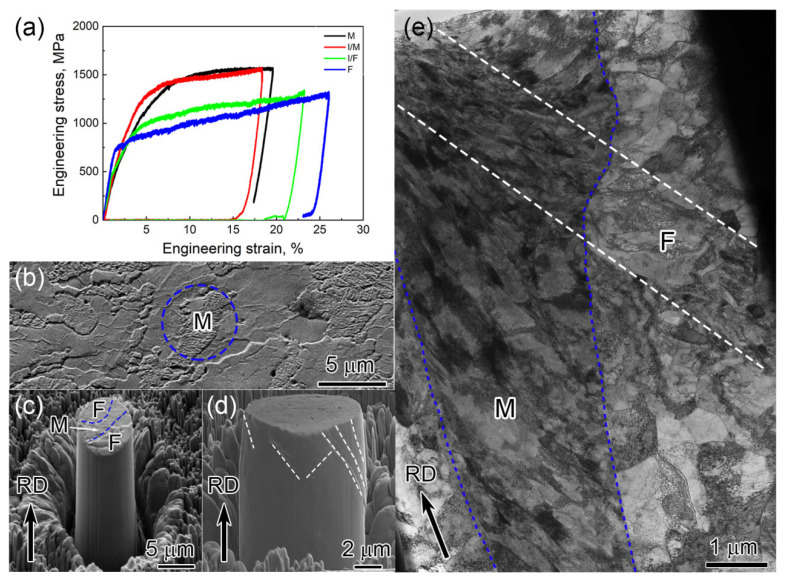
(**a**) Engineering stress–strain curves of the micropillars containing the single ferrite phase, martensitic phase and only one F/M interphase. (**b**) SEM image of the top view of the F/M/F pillar before FIB milling (RD surface). (**c**) SEM image of the micropillar before compression and (**d**) after compression. (**e**) TEM bright field image of the compressed micropillar’s cross-section, obtained by FIB lift-out from (**d**).

Figure 3a shows the engineering stress–strain curves of micropillars compressed on the single ferritic phase, martensitic phase, and F/M interfaces. At the early stage of deformation, all pillars follow the linear elastic deformation. The single-ferrite-pillar yield is about 730 MPa, while the other pillars have an elastoplastic deformation character where the yielding point is hard to determined. The transition between elastic deformation to plastic deformation starts from approx. 500 MPa to approx. 1500 MPa for the martensite single-phase (M) pillar and the pillar containing high-volume martensite (I/M, VM of about 65%) pillar. The pillar with a high-volume fraction of F (I/F, VM of about 25%) has a UTS slightly larger than that of the single-ferrite-phase (F) pillar. Representative in situ compression of an F/M interfaces micropillar is shown in Figure 3b–e. The pillar, as shown in Figure 3c, was milled from the area indicated by the blue circle in Figure 3b, where the martensite laminate was confined by two ferrite lamellae. As shown in Figure 3d, at least two slip systems are identified to be activated during compression. Figure 3e reveals the TEM bright field image of the cross-section of the compressed micropillar. It is found that the martensite laths at the pillar top are highly distorted and different from those aligned at the bottom of the pillar (such as below the mark “M”). A slip/shear band across martensite and ferrite are indicated by white lines, indicating synergistic deformation of martensite and ferrite during compression. It is believed that slip/shear bands were formed during compression and these bands extended from ferrite to martensite (or verse versa). The overall deformation applied on the micropillar was coordinated by local slip/shear of martensite and ferrite, where slip/shear bands formed continuously across the ferrite/martensite interface. Therefore, a synergistic deformation of the ferrite and martensite phases can be deduced in the dual-phase steel. It was reported that if the block boundary of martensite exhibited a high resolved shear stress, sliding would occur along martensite block boundaries and thus enhance the plasticity of martensite [18,19]. Cavusoglu et al. demonstrated via the 3D representative volume element (RVE) method that an increase in martensite particle size leads to a reduction in tensile strength accompanied by an increase in deformation amount [20]. Compared with the hot-rolled samples with an engineering strain of 63% [10], the excellent mechanical properties of the steel are attributed to the refinement of ferrite/martensite layers and ultra-fine martensite laths. However, the present result demonstrates that the plastic deformation of low-carbon martensite can also be realized by coordination between martensite and ferrite.

**Figure 4:**
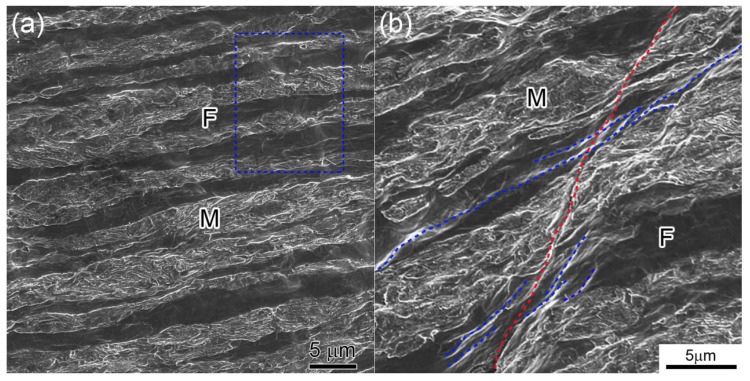
SEM images of the tensile tested HR-98% sample (**a**), where (**b**) is closer to the fracture surface.

To verify this mechanism, the microstructures of macroscopic tensile samples were investigated (as shown in Figure 4a). Figure 4b was acquired from a region adjacent to the fracture surface, where the engineering strain was approximately 9.4%. It is widely recognized that plastic deformation firstly takes place in the soft ferrite phase while the hard martensite phase remains in an elastic state. With increasing plastic strain, internal stress will build up at the ferrite/martensite interfaces and when the applied stress is high enough to initiate deformation in the martensite phase, the martensite phase starts to deform plastically [21].

Due to the large difference in deformation ability, the stress–strain curve of HR-98% revealed an elastoplastic character before obvious plastic deformation, as shown in Figure 2a. At the same time, as shown in Figure 4a, slip/shear bands are formed in ferrite. When these slip bands extend to the ferrite/martensite boundary, they will be stopped by the hard martensite phase. Most of the cases in dual-phase steel are that micro-voids may be formed at these stress-intensive locations [2], since the stress cannot be released by dislocation–boundary interactions. However, in the present HR-98% sample, synergistic deformation of the ferrite and martensite phases occurred instead of formation of micro-voids, as shown by a red line in Figure 4b where a continuous band extended several ferritic and martensitic layers. Similar bands can be also observed, formed at earlier stages of deformation, marked in blue rectangles in Figure 4a. These synergistic deformations in both ferrite and martensite are believed to be the main reason for good ductility of the lamellae dual phase [22,23,24]. Due to preferential activation of in-lath-plane slip in martensite, along with a high population of dislocations that can glide parallel to the lath interface with a long mean free path, the ductility of martensite is increased [22,24]. In addition, the interface effect between martensite and ferrite as revealed in the steel after 63% hot-rolling may also be active, which is beneficial to ductility and strength [10,23].

## 4. Conclusions

In summary, the mechanical behaviors of ferrite and martensite in a laminated dual-phase 0.1C5Mn3Al steel are revealed by nano-indentation and in situ micropillar compression in SEM.

(1) Both hot-rolling and cold-forged samples show a laminated/layered structure characterized by a ferrite phase and martensite phase alternatively distributed perpendicular to the rolling direction (RD), and the volume fractions of martensite are both close to 60%.

(2) For the hot-rolling samples subjected to 98% strain, the ferrite layers consist of equiaxed dislocation cells and sub-grains with an average size of 540 nm, whereas the martensite lamellae are composed of ultra-fine martensite laths with an average thickness of 200 nm. Corresponding to this microstructure, the HR-98% samples exhibit an ultimate tensile strength (UTS) of 1277 ± 44 MPa and a total elongation (TE) of 11.8 ± 0.4%.

(3) It is found that extended slip/shear bands were formed in the ferrite and martensite phases during deformation. Synergistic deformation was achieved via slip of martensite phases which resulted in a good combination of strength and ductility of the 98% hot-rolled samples.

## Data Availability

The original contributions presented in this study are included in the article. Further inquiries can be directed to the corresponding author.
